# Molecular delineation of small supernumerary marker chromosomes using a single nucleotide polymorphism array

**DOI:** 10.1186/s13039-020-00486-2

**Published:** 2020-05-27

**Authors:** Lili Zhou, Zhaoke Zheng, Lianpeng Wu, Chenyang Xu, Hao Wu, Xueqin Xu, Shaohua Tang

**Affiliations:** 1 Center of Prenatal Diagnosis, Wenzhou Central Hospital, Wenzhou, 325000 People’s Republic of China; 2 Key laboratory of Medical Genetic, School of Laboratory Medicine and Life Science, Wenzhou Medical University, Wenzhou, 325000 People’s Republic of China

**Keywords:** Small supernumerary marker chromosome, Single nucleotide polymorphism array, Prenatal diagnosis

## Abstract

**Background:**

Defining the phenotype-genotype correlation of small supernumerary marker chromosomes (sSMCs) remains a challenge in prenatal diagnosis. We karyotyped 20,481 amniotic fluid samples from pregnant women and explored the molecular characteristics of sSMCs using a single nucleotide polymorphism (SNP) array.

**Results:**

Out of the 20,481 samples, 15 abnormal karyotypes with sSMC were detected (frequency: 0.073%) and the chromosomal origin was successfully identified by SNP array in 14 of them. The origin of sSMCs were mainly acrocentric-derived chromosomes and the Y chromosome. Two cases of sSMC combined with uniparental disomy (UPD) were detected, UPD(1) and UPD(22). More than half of the cases of sSMC involved mosaicism (8/15) and pathogenicity (9/15) in prenatal diagnosis. A higher prevalence of mosaicism for non-acrocentric chromosomes than acrocentric chromosomes was also revealed. One sSMC derived from chromosome 3 with a neocentromere revealed a 24.99-Mb pathogenic gain of the 3q26.31q29 region on the SNP array, which presented as an abnormal ultrasound indicating nasal bone hypoplasia.

**Conclusion:**

The clinical phenotypes of sSMCs are variable and so further genetic testing and parental karyotype analysis are needed to confirm the characteristics of sSMCs. The SNP array used here allows a detailed characterisation of the sSMC and establishes a stronger genotype-phenotype correlation, thus allowing detailed genetic counselling for prenatal diagnosis.

## Background

Small supernumerary marker chromosomes (sSMCs) are structurally abnormal chromosome fragments that cannot be clearly determined by conventional banding cytogenetics alone and are equal in size or smaller than a chromosome 20 of the same metaphase spread [1]. Approximately 77% of sSMCs are de novo and 23% are inherited, either maternally (16%) or paternally (7%) [2]. Approximately one-third of sSMC cases are associated with specific clinical symptoms, (e.g. i(12p) and Pallister–Killian syndrome, sSMC(15) and Prader–Willi Syndrome/Angelman Syndrome (PWS/AS), and inv. dup(22) and cat-eye syndrome) while two-thirds of sSMC cases have not been associated with clinical syndromes [3].

sSMCs are found in 0.072–0.075% of prenatal cases and 0.044% of live births, although the rate is increased to 0.288% in patients with intellectually disabled patients [4, 5]. Defining the phenotype-genotype correlation of sSMCs remains a challenge due to their complex origins and genetic materials. The phenotype of sSMCs are variable and include mental and growth retardation, craniofacial and urogenital abnormalities, and cardiac anomalies, which are associated with the size of the sSMCs, gene content, mosaicism percentage, uniparental disomy, and other concomitant imbalances [6]. Chromosomal microarray analysis is a sensitive technique for characterising sSMCs and can not only detect genomic copy number changes but also define breakpoints and the genes involved [7–9].

Here, we studied 15 cases of sSMCs through cytogenetic and SNP array analyses and provide a meaningful genotype-to-phenotype correlation for genetic counselling.

## Results

### Frequency and incidence of parentally inherited sSMCs

A total of 15 cases of sSMCs initially were detected from 20,481 samples of amniotic fluid by the conventional karyotype analysis with and an overall frequency of 0.073%. The parental karyotype was investigated, and three cases were parentally inherited, of which 2/15 were maternally inherited and 1/15 was paternally inherited.

### Chromosomal origin and pathogenicity of sSMCs by SNP array

All 15 cases were subjected to SNP array and the chromosome origin was successfully identified 14 of them. The reason for the remaining negative SNP array result is uncertain but it may be due to the sSMC being derived from an acrocentric chromosome and containing only centromere heterochromatin. The foetus inherited the sSMC from his phenotypically normal mother. Due to the limited amount of specimen, no further study was performed.

**Fig. 1:**
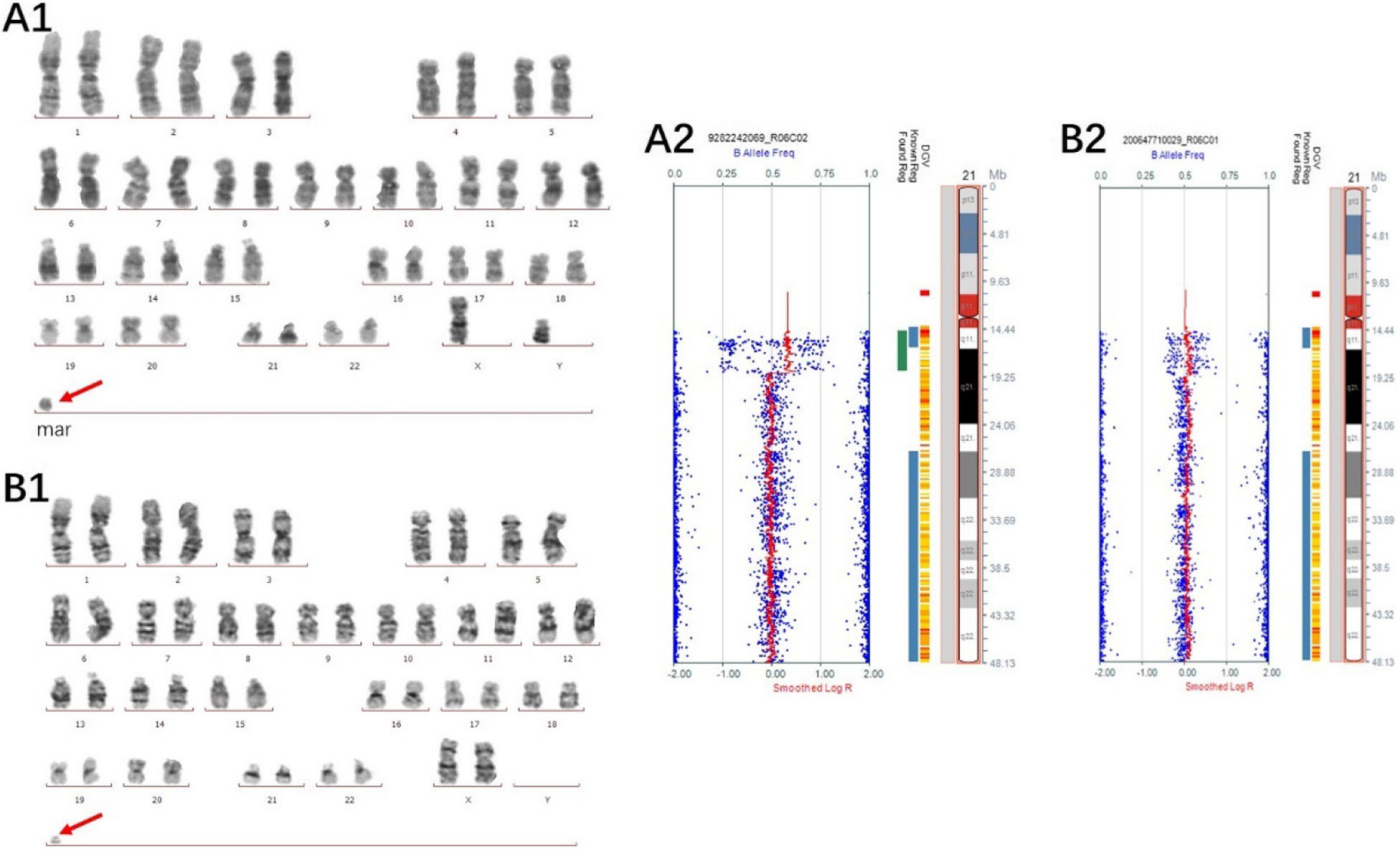
Cytogenetic and SNP array results of the sSMC derived from chromosome 21. (A1) G-banding of case 9 revealed the karyotype 47,XY,+mar[14]/46,XY[16]. (A2) The sSMC of case 9 was a mosaic partial tetraploid of chromosome 21: arr 21q11.2–21.1(14687571–18864186)×2~4. (B1) G-banding of the mother (Case 9) revealed the karyotype 47,XX,+mar[11]/46,XX[39]. (B2) The sSMC of the mother was a partial duplication of chromosome 21: arr 21q11.2q21.1(14687571-18864186)×2~3

**Fig. 2:**
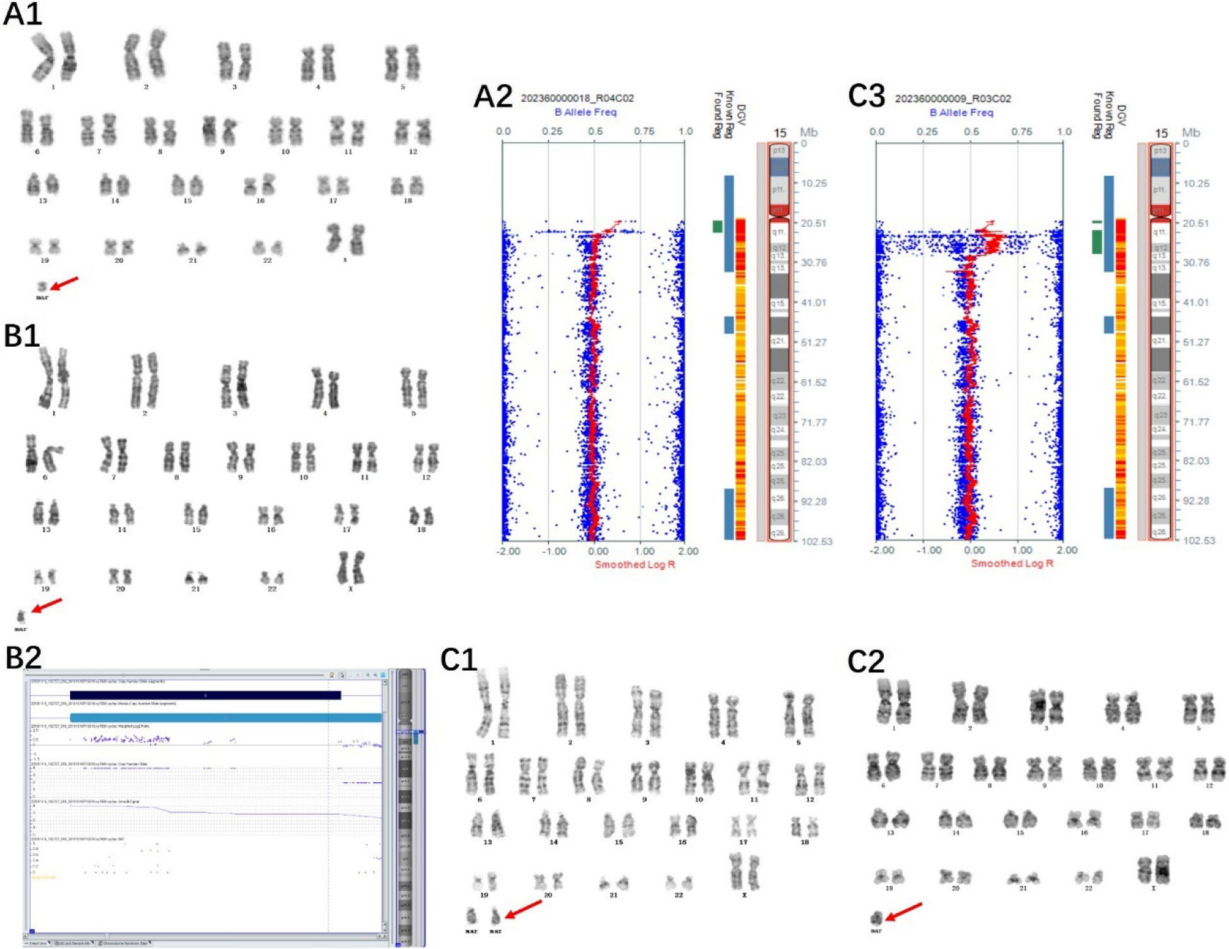
Cytogenetic and SNP array results of sSMCs derived from chromosome 15. (A1) G-banding of case 5 revealed the karyotype 47,XX,+mar in all cells. (A2) The sSMC of case 5 was a partial tetraploid: arr 15q11.1q11.2(20161372_23300172)×4. (B1) G-banding of case 6 revealed the karyotype 47,XX,+mar in all cells. (B2) The sSMC of case 6 was a partial tetraploid: arr 15q11.2(22770421_23625785)×4. (C1, C2) G-banding of case 7 revealed the karyotype 48,XX,+2mar[30]/47,XX,+mar[28]/46,XX[7]. (C3) The sSMC of case 7 was a mosaic partial hexaploid: arr 15q11.2q13.1(22754322_28969665)×2~6

**Fig. 3:**
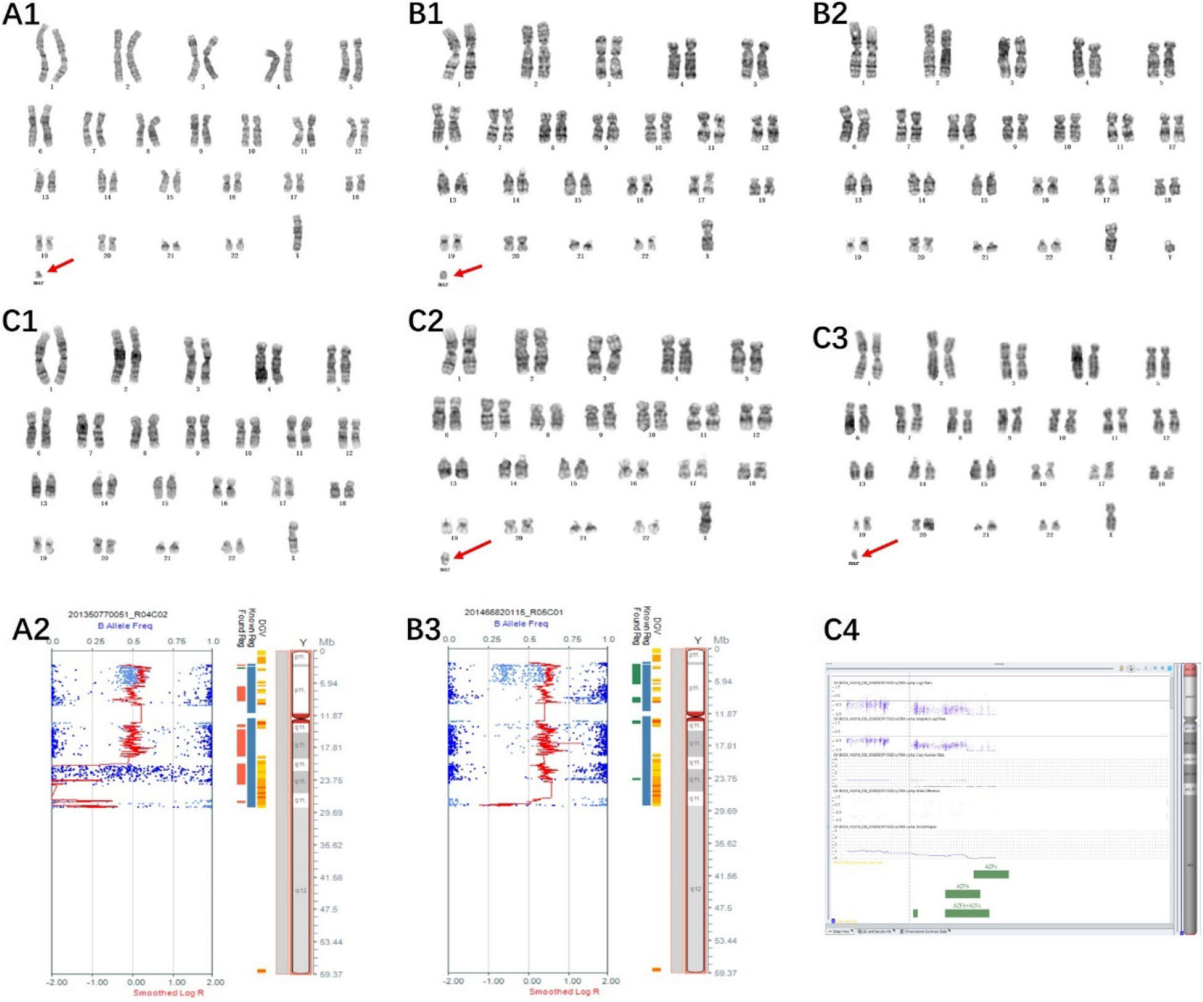
Cytogenetic and SNP array results of sSMCs derived from the Y chromosome. (A1) G-banding of case 13 revealed the karyotype 46,X,+mar in all cells. (A2) The sSMC of case 13 was a partial deletion of the Y chromosome: arr Yq11.222q11.23(20828795_28799654)×0. (B1, B2) G-banding of case 14 revealed the karyotype 46,X,+mar[17]/46,XY[15]. (B3) The sSMC of case 14 was a partial duplication and deletion of the Y chromosome: arrYp11.31q11.23(2655180_28498354)×2, Yq11.23(28455408_28760588)×0. (C1, C2, C3) G-banding of case 15 revealed the karyotype 45,X[14]/46,X,+mar1[9]/46,X,+mar2[6]. (C4) The sSMC of case 15 was a mosaic partial deletion of the Y chromosome: arr Yq11.21q11.223(13800955_23653757)×0~1,Yq11.223q11.23(23653757_28799654)×0

**Fig. 4:**
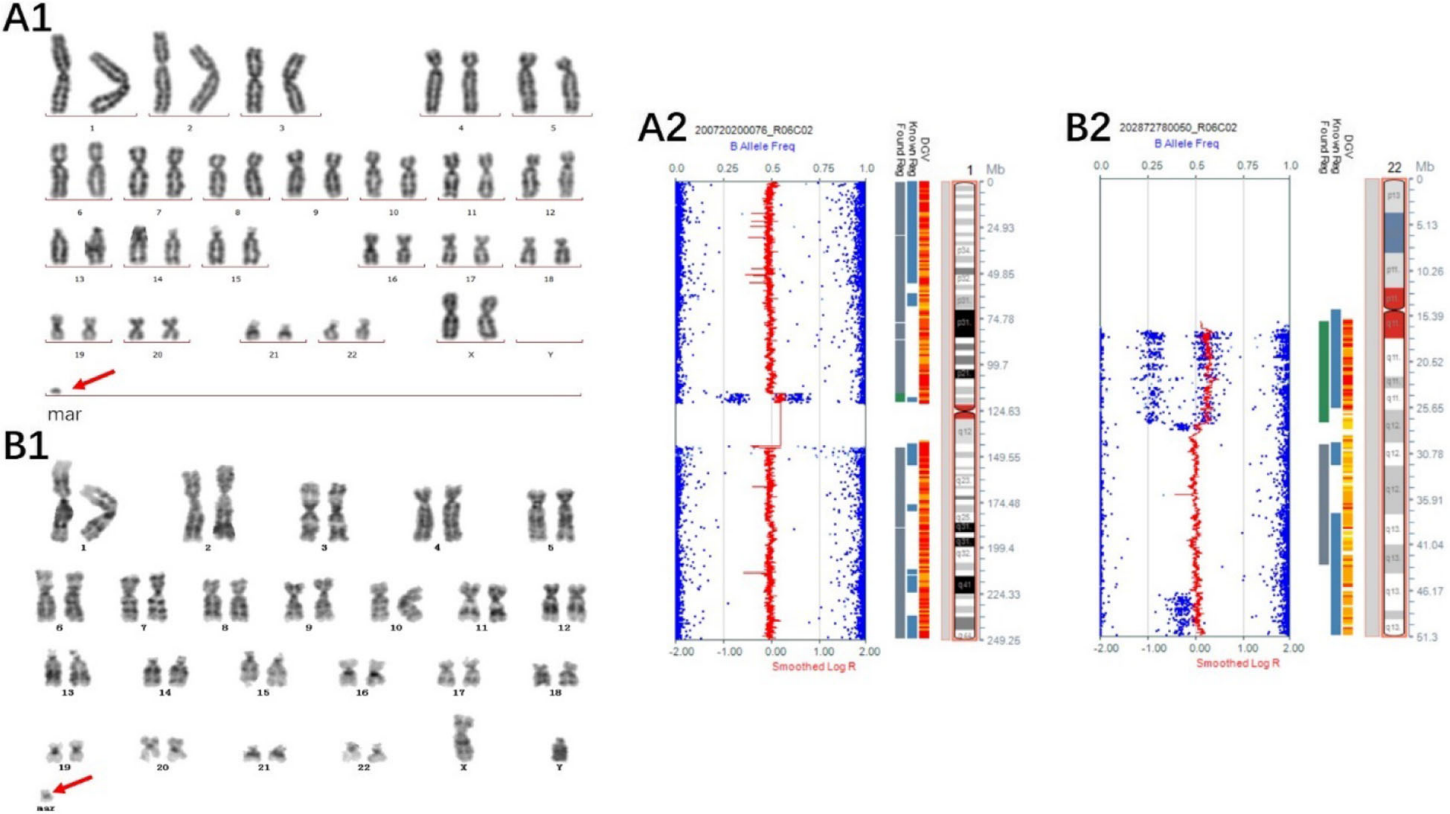
Cytogenetic and SNP array results of sSMCs combined with UPD. (A1) G-banding of case 1 revealed the karyotype 47,XX,+mar[53]/46,XX[22]. (A2) The sSMC of case 1 was a partial duplication of chromosome 1 combined with UPD(1): arr 1p13.2p11.2(115796490–121184898)×3, UPD(1). (B1) G-banding of case 11 revealed the karyotype 47,XY,+mar. (B2) The sSMC of case 11 was a partial duplication of chromosome 22 combined with UPD(22): arr 22q11.1q12.1(16079545–27421632)×3, 22q12.2q13.2(29841642–43483242)×2 hmz

**Fig. 5:**
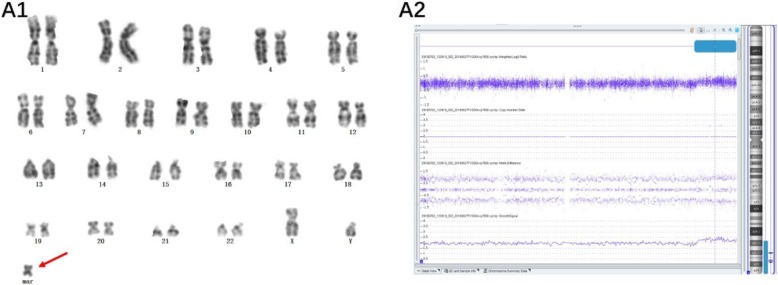
Cytogenetic and SNP array results of neocentric sSMC(3). (A1) G-banding of case 3 revealed the karyotype 47,XY,+mar[7]/46,XY[38]. (A2) The sSMC of case 3 was a mosaic partial duplication of chromosome 3: arr 3q26.31q29(172862469_197851444)×2~3[30%]

Of the 14 cases with chromosome gain detected by the SNP array, one sSMC was derived from each of chromosome 1 (Case 1), chromosome 2 (Case 2), chromosome 3 (Case 3), and chromosome 4 (Case 4), and one isochromosomal sSMC was derived from each of chromosome 18 (Case 8) and chromosome 21 (Case 9) (Fig. 1). Three sSMCs originated from chromosome 15 (Case 5, Case 6 and Case 7) and only one (Case 7) contained the PWS/AS critical region (15q11q13) (Fig. 2). Two sSMCs originated from chromosome 22 (Case 10 and Case 11) and three cases derived from the Y chromosome (Case 13, Case 14 and Case 15) (Fig. 3). In summary, 21% of cases originated from chromosome 15, 21% from acrocentric chromosomes other than chromosome 15, 37% from non-acrocentric chromosomes and 21% from the Y chromosome. Two cases of sSMC combined with UPD were detected (2/15), UPD(1) and UPD(22) (Fig. 4). One sSMC derived from chromosome 3 with a neocentromere revealed a 24.99 Mb pathogenic gain of the 3q26.31q29 region [arr 3q26.31q29(172862469_197851444)×2~3[30%]], which presented as an abnormal ultrasound indicating nasal bone hypoplasia (Fig. 5).

Of the 14 cases of sSMCs, 64% (9/14) were pathogenic or likely pathogenic, 29% (4/14) were a variant of unknown significance and 7% (1/14) were likely benign. The results of the karyotyping and the SNP array are summarised in Table 1.

**Table 1:** Summary of karyotypes, SNP array result, ultrasound findings and pregnancy outcome

**Case no.**	**Karyotype**	**SNP array**	**Size (Mb)**	**PC**	**Indication**	**De novo/inherited**	**Ultrasound findings**	**Pregnancyoutcome**
1	47,XX,+mar[53]/46,XX[22]	1p13.2p11.2(115796490-121184898)×3,	5.39	VOUS	high risk of T21(1/230)	de novo	no abnormal changes	termination of pregnancy
		UPD(1)	/	VOUS				
2	47,XX,+mar[6]/46,XX[24]	2p12q12.1(82947063-104957361)×2~3[75%]	22.01	VOUS	oligohydramnios	de novo	oligohydramnios	termination of pregnancy
3	47,XY,+mar[7]/46,XY[38]	3q26.31q29(172862469_197851444)×2~3[30%]	24.99	P	Abnormal pregnancy history	de novo	nasal bone hypoplasia	termination of pregnancy
4	47,XY,+mar[30]/46,XY[9]	4p12q13.1(46214008_60186304)×3	13.97	LP	high risk of T21(1/147)	de novo	no abnormal changes	termination of pregnancy
5	47,XX,+mar	15q11.1q11.2(20161372_23300172)×4	3.14	VOUS	advanced maternal age	de novo	NT4.8mm	Continued gestation
6	47,XX,+mar	15q11.2(22770421_23625785)×4	0.86	VOUS	high risk of T21(1/220)	de novo	/	Continued gestation
7	48,XX,+2mar[30]/47,XX, +mar[28]/46,XX[7]	15q11.2q13.1(22754322_28969665)×2~6	6.22	LP	advanced maternal age	de novo	/	termination of pregnancy
8	47,XY,+mar	18p11.32p11.21(12842_15375878)×4	15.36	P	high risk of T18(NIPT)	de novo	/	termination of pregnancy
9	47,XY,+mar[14]/46,XY[16]	21q11.2q21.1(14687571-18864186)×2~4	4.18	LB	high risk of T21(1/180)	mat	no abnormal changes	Continued gestation
10	47,XY,+mar	22q11.21(18648855_21464764)×4	2.82	LP	NT3.4mm	de novo	pyelectasis	Continued gestation
11	47,XY,+mar	22q11.1q12.1(16079545_27421632)×3,	11.34	LP	high risk of T22(NIPT)	de novo	no abnormal changes	termination of pregnancy
		22q12.2q13.2(29841642_43483242)×2hmz	/	VOUS				
12	47,XX,+mar	normal	/	/	advanced maternal age	mat	no abnormal changes	Continued gestation
13	46,X,+mar	Yq11.222q11.23(20828795_28799654)×0	7.97	P	advanced maternal age	pat	no abnormal changes	Continued gestation
14	46,X,+mar[17]/46,XY[15]	Yp11.31q11.23(2655180_28498354)×2,	25.84	VOUS	advanced maternal age	de novo	no abnormal changes	termination of pregnancy
		Yq11.23(28455408_28760588)×0	0.31	P				
15	45,X[14]/46,X,+mar1[9]/46,X,+mar2[6]	Yq11.21q11.223(13800955_23653757)×0~1,	9.85	P	high risk of chr X(NIPT)	de novo	no abnormal changes	termination of pregnancy
		Yq11.223q11.23(23653757_28799654)×0	5.15	P				

*PC*, pathogenicity classification, *VOUS* variant of unknown significance, *P* pathogenic, *LP* likely pathogenic, *LB* likely benign

### Chromosomal origin distribution of mosaic sSMCs

Cytogenetic analysis showed mosaic sSMCs in 8/15 cases (Case 1, Case 2, Case 3, Case 4, Case 7, Case 9, Case 14 and Case 15) with the sSMC cell line mosaic level ranging from 16 to 77%. The frequencies of mosaic sSMCs derived from non-acrocentric, acrocentric and Y chromosomes were 80% (4/5), 33% (2/6) and 67% (2/3), respectively.

### Correlation between the indications for invasive prenatal diagnosis and the presence of sSMCs

In our survey, seven cases of sSMC were referred due to positive serological screening (7/15), four cases due to ultrasound anomalies (4/15) and four cases due to advanced maternal age (4/15). The low incidence of sSMCs in women referred due to ultrasound anomalies may be biased because pregnant women with ultrasound anomalies may have opted for a termination, whereas those without obvious anomalies by ultrasound testing likely opted to continue the pregnancy.

## Discussion

In the present survey, we described 15 sSMCs involving different chromosomes with a frequency of 0.073% in 20,481prenatal diagnosis cases, which was in agreement with the incidence of 0.075% previously reported [4]. The origins of sSMCs in our survey were derived from acrocentric chromosomes (42%), followed by non-acrocentric chromosomes (37%) and the Y chromosome (21%), which is in agreement with the literature [10]. It is reported that chromosome 15 was the most common origin for sSMCs in acrocentric chromosomes, accounting for ~ 30–50% [11]. Chromosome 15-derived sSMCs incorporating the PWACR are associated with developmental delay, mental retardation, ataxia, seizures and behavioural problems and patients with more copies of this region may develop a more severe phenotype [12]. In contrast, sSMCs(15) without the PWACR has no or only a minor influence on the carrier’s phenotype but is associated with a high incidence of infertility in males [13]. Patients that are positive for sSMC(15) even without the involvement of the PWACR should also undergo prenatal testing for UPD(15) [14]. In our study, three cases of sSMCs originated from chromosome 15 in total. The sSMC of case 7 included four to six copies of the PWACR and was evaluated as pathogenic; the couple decided to terminate the pregnancy. The sSMCs of cases 5 and case 6 did not involve the PWACR and were evaluated as a variant of unknown significance. Two couples continued the pregnancies and the newborns did not display any clinical phenotype at 9 or 15 months. No UPD conditions for imprinted chromosome 15 were found in these three cases.

Routine cytogenetic analysis showed mosaic sSMCs in 8 out of the 15 cases, with the sSMC cell line mosaic level ranging from 16 to 77%. sSMCs are quite unstable during mitosis leading to mosaicism, which is estimated to be present in 50% of sSMC cases, which is in accordance with our data [15]. The frequency of mosaic sSMCs derived from non-acrocentric, acrocentric and Y chromosomes was 80% (4/5), 33% (2/6) and 67% (2/3), respectively. A higher prevalence of mosaicism in non-acrocentric chromosomes than acrocentric chromosomes may be related to dose sensitivity. Liehr and Al-Rikabi found that mosaicism was the reason for the normal phenotypes in carriers of sSMC with known and well-defined syndromes. In 2% of cases, there was a normal or much less severe outcome than expected in low-mosaic samples [16].

Only around 30% of sSMC carriers are clinically impaired after birth and the pathogenicity of sSMCs is higher in prenatal cases [17]. In our survey, 14 cases of sSMC were successfully identified by SNP array and nine cases were pathogenic or likely pathogenic (9/14), four cases were a variant of unknown significance (4/14) and one case was likely benign (1/14). We speculated that sSMCs with a clinical phenotype would more likely lead to a termination of the pregnancy, thus decreasing the frequency of pathogenicity in live births. For the nine cases of pathogenicity or likely pathogenicity, seven pregnancies were terminated and two were continued. Case 10 presented with a karyotype of 47,XY,+mar with an increased nuchal translucency of 3.4 mm. A small marker chromosome was tetraploid for 2.82 Mb in the region 22q11.21(chr22: 18,648,855_21,464,764). This region correlates with cat-eye syndrome, which is a rare genetic syndrome with an incidence of around 1/150,000 live births and is caused by partial tetrasomy of chromosome 22 [18]. The classical triad of CES consists of iris colobomas, anal malformations and ear anomalies. Ultrasound findings of case 10 indicated pyelectasis during the second trimester. The pregnancy was continued and the mother gave birth to a boy at 39 + 2 weeks gestation (birth weight 2990 g, length 50 cm and head circumference 34 cm). Apgar scores were 10 (1′) and 10 (5′). Neonatal pneumonia was diagnosed 1 h after birth with arterial blood gas results of pH 7.329, PCO_2_ 33.7 mmHg and PO_2_ 37 mmHg.

Case 13 also showed a pathogenic sSMC but continued gestation. The karyotype was 46,X,+mar and showed a loss of 7.97 Mb for the region Yq11.222q11.23 (chrY: 20,828,795_28,799,654), which was a deletion of the Y chromosome in the AZFb+AZFc region. The father was also a carrier of an sSMC with the same morphology and presenting oligozoospermia. The AZF region that undergoes microdeletions has been mapped to Yq11.22–23 and consists of three subregions called AZFa, AZFb and AZFc, which are associated with male oligo/azoospermia and accounts for 10–12% of phenotypically normal men with idiopathic infertility [19]. The most frequent deletion type is of the AZFc region (~ 80%) followed by AZFa (0.5–4%), AZFb (1–5%) and AZFb+c (1–3%) [20]. Deletions of the entire AZFa region are associated with an invariable clinical phenotype of Sertoli cell-only syndrome and azoospermia, whereas deletions of the AZFc region are compatible with residual spermatogenesis and severe oligozoospermia may even be transmitted naturally to the male offspring in rare cases [21]. Furthermore, there is a ~ 50% chance of retrieving spermatozoa by testicular sperm extraction and conceiving children by intracytoplasmic sperm injection for men with azoospermia and AZFc deletion [22]. AZFb+c deletions are similar to the complete deletions of the AZFa region; however, spermatid arrest and even crypto/oligozoospermia have been reported in only three cases [23, 24]. Case 13 chose to continue the pregnancy and a boy was born naturally at 39 + 4 weeks. The boy was followed-up to 1 year and his development and growth were normal.

Approximately 77% of sSMCs are de novo while 23% are inherited, either maternally (16%) or paternally (7%) [2]. In our study, three cases were inherited, two of which maternally and one paternally. Case 9 was at high risk of trisomy 21(1/180) and presented with a mosaic karyotype of 47,XY,+mar[14]/46,XY[16]. The sSMC was close to the centromere and included a tetraploid gain of 4.18 Mb of the 21q11.2q21.1 region, which is a dose-insensitive region and does not contain the Down syndrome critical region (DSCR). Duplication of the DSCR due to duplication of 0.6–8.3 Mb within human chromosome 21q22 is sufficient to induce the major phenotypes of Down syndrome, i.e. mental retardation, congenital heart disease, characteristic facial appearance, and probably the hand and dermatoglyphic abnormalities [25]. Patients excluding the DSCR present mild and non-specific phenotypes, such as joint hyperlaxity, hypotonia and brachycephaly hypertelorism, epicanthic folds, strabismus and mildly dysmorphic ears [26]. The mother of case 9 without any abnormal clinical phenotype was also a carrier of an sSMC with a mosaic karyotype of 47,XX,+mar[11]/46,XX[39], which is a triploid gain for the same region of 21q11.2q21.1 but with a different morphology, suggesting that the sSMC underwent recombined duplication during through two generations. If the additional euchromatic material is one small copy near the centromere, it can be tolerated. Whereas if the additional euchromatic material is too large or involves between four and six copies, an abnormal phenotype occurs [17]. We estimated the sSMC as likely a benign variant and would have no or only a minor influence on the carrier’s phenotype, thus the women chose to continue the pregnancy. A boy was born naturally at 39 + 6 weeks and was followed-up for 3 years with development and growth both normal.

Another consequence of sSMC is the risk for uniparental disomy and 1.3% of sSMC cases present with UPD [27, 28]. In cases with UPD, 40 of 46 cases (87%) of cases had a maternal UPD and only 13% were paternal [29]. Thus far, only five chromosomes have been defined as imprinted based on the associated clinical phenotypes: chromosomes 6, 7, 11, 14 and 15. In our study, two cases of sSMC were combined with UPD (UPD(1) and UPD(22)). Chromosomes 1 and 22 are concerned imprinting disease impossibility, but the potential of unmask recessive alleles has been described for several diseases [30]. Case 1 had UPD for the entire chromosome 1. The foetus, therefore, was at increased risk for recessive genetic diseases and rare disorders [31]. We deemed the sSMC and UPD(1) to both be a variant of unknown significance and the formation was likely via trisomy rescue. For case P11, the SNP array detected an additional chromosome abnormality, i.e. a loss of heterozygosity at segmental chromosome 22q12.2q13.2(chr22: 29,841,642_43,483,242). Six clinically normal cases with UPD(22) mat and three cases of recessive gene activation due to UPD(22) mat have been reported [28]. We classified the sSMC as likely pathogenic and UPD(22) as a variant of unknown significance. These data suggested that some sSMC patients may have additional chromosomal UPD anomalies and thus could be underestimated without advanced molecular techniques.

Case 3 presented an abnormal ultrasound indicating nasal bone hypoplasia and a mosaic karyotype of 47,XY,+mar[7]/46,XY[38]. The sSMC was a 24.99 Mb gain of the 3q26.31q29 region [arr 3q26.31q29(172862469_197851444)×2~3[30%]], with a neocentromere. Neocentric sSMCs constitute one of the smallest groups of reported sSMCs and have a centromeric constriction but without detectable alpha-satellite DNA [32]. Neocentric sSMCs carry newly derived centromeres that are apparently formed within interstitial chromosomal sites and have no centromeric function, but it is unclear how the neocentromere is acquired and formed on an acentric fragment [33]. More than 90% of cases of a neocentric sSMC are associated with an adverse clinical outcome, which is mainly due to the size of the imbalanced chromosome dosage. In total, 10 neocentric sSMCs of chromosome 3 have been reported, nine of which were derived from the distal tip of the long arm [17]. Typical phenotypes of neocentric sSMC(3) are dysmorphic features (64%), streaky hyperpigmentation (55%), mental retardation (45%), kidney problems (36%, and polydactyly (27%) [28]. We classified the sSMC of case 3 as pathogenic, thus the pregnancy was terminated.

The shortcoming of this paper was that interphase fluorescence in situ hybridisation (FISH) analyses were not applied to identify the morphology due to a limited amount of specimens and time in prenatal diagnosis, which was not indispensable for pathogenicity assessment and genetic counselling. The disadvantage of chromosomal microarray analysis for sSMC characterisation is if the sSMC contains only heterochromatin, it may not be identified. In this study, the failure to detect the chromosomal origin of cases 12 is likely due to the same reason. Meanwhile, low-level mosaicism (< 20%) can also be missed. The combined application of traditional and molecular cytogenetic analyses has a critical role in precisely characterising sSMCs, including the mosaic form, molecular components, and shape of the sSMCs, which offers more information for genetic counselling.

## Conclusion

We identified 15 sSMCs by SNP array analyses which allows a detailed characterisation of the sSMC and establishes a stronger genotype-phenotype correlation, thus allowing detailed genetic counselling for prenatal diagnosis.

## Methods

### Study subjects

This study was approved by the institutional research ethics committee of Wenzhou Central Hospital. All parents agreed to participate in the study and provided written informed consent. We retrospectively analysed a cohort of 20,481 amniotic fluid samples that were taken at the Wenzhou Prenatal Diagnosis Center between 2014 and 2019. The pregnant women ranged in age from 19 to 48 years, with their gestational week between 16 and 24 weeks. The indications for prenatal diagnosis included advanced maternal age, high-risk serological screening, abnormal non-invasive prenatal DNA test, ultrasonographic abnormal indications, either parent carrying a chromosome abnormality, and a history of intrauterine foetal death or aborted foetuses. Peripheral venous blood was collected from the parents if necessary.

### Karyotype analysis

A total of 20,481 foetal amniotic fluid samples were analysed using standard G-banded karyotyping at 320–450 band resolution to diagnose sSMCs.

For culture cells, 20 ml of amniotic fluid was centrifuged at 1200 rpm for 10 min. The supernatant was discarded after centrifugation, leaving about 1–2 ml of cell suspension. Amniocyte culture medium (5 ml) was added and cells were grown in an incubator at 37 °C and 5% CO_2_ for 9 to 10 days.

For karyotyping, cells were harvested in situ cultures and conventional G-banding was performed, and then cells were scanned by a Leica GLS120 automated nuclear scanning system. Fifteen chromosome karyotypes were counted and five karyotypes were analysed by two doctors according to the International System for Human Cytogenetic Nomenclature 2016. Karyotyping was also performed on parental blood samples to determine whether the sSMC detected in the foetal samples was inherited or de novo.

### SNP array analysis

Chromosomal microarray analysis was performed on the 15 cases of sSMC using the Affymetrix CytoScan 750 k Array or the Illumina Human CytoSNP-12 array according to the manufacturer’s instructions. The results were analysed with Chromosome Analysis Suite software or Illumina’s BeadStudio software. All detected CNVs were compared with known CNVs in the scientific literature and with those in the following publicly available databases: Database of Genomic Variants (http://dgv.tcag.ca/dgv/app/home), DECIPHER database (http://decipher.sanger.ac.uk/), International Standards for Cytogenomic Array (ISCA; https://www.iscaconsortium.org/), Online Mendelian Inheritance in Man (OMIM; http://www.omim.org) and ClinGen Dosage Sensitivity Map (https://www.ncbi.nlm.nih.gov/projects/dbvar/clingen).

Based on the American College of Medical Genetics Standards and Guidelines, the CNVs were classified as pathogenic (P), likely pathogenic (LP), likely benign (LB), variant of unknown significance (VOUS) or benign (B). All reported CNVs were based on the National Center for Biotechnology Information human genome build 37 (hg 19).

## Data Availability

All data generated or analyzed during this study are included in the article.

## Abbreviations

sSMC: Small supernumerary marker chromosome
SNP: Single nucleotide polymorphism
UPD: Uniparental disomy
PWS: Pallister Killian syndrome
AS: Angelman Syndrome
PWACR: Prader-Willi Syndrome/Angelman Syndrome critical region
CES: Cat-eye syndromes
DSCR: Down syndrome critical region
FISH: Interphase fluorescence in situ hybridization
